# A Chilean Berry Concentrate Protects against Postprandial Oxidative Stress and Increases Plasma Antioxidant Activity in Healthy Humans

**DOI:** 10.1155/2017/8361493

**Published:** 2017-01-24

**Authors:** Ines Urquiaga, Felipe Ávila, Guadalupe Echeverria, Druso Perez, Sebastian Trejo, Federico Leighton

**Affiliations:** ^1^Center for Molecular Nutrition and Chronic Diseases, Pontificia Universidad Católica de Chile, Casilla 114-D, Santiago, Chile; ^2^Escuela de Nutrición y Dietética, Facultad de Ciencias de la Salud, Universidad de Talca, 3460000 Talca, Chile

## Abstract

This study formulated and characterized an antioxidant-rich concentrate of berries (BPC-350) produced in Chile, which was used to perform a crossover study aimed at determining the effect of the berries on the modulation of plasma postprandial oxidative stress and antioxidant status. Healthy male volunteers (*N* = 11) were randomly assigned to three experimental meals: (1) 250 g of ground turkey burger (GTB) + 500 mL of water; (2) 250 g of GTB + 500 mL of 5% BPC-350; (3) 250 g of GTB prepared with 6% BPC-350 + 500 mL of 5% BPC-350. Venous blood samples were collected prior to meal intake and every hour for six hours after intake. Malondialdehyde (MDA), carbonyls in proteins, and DPPH (2,2-diphenyl-1-picrylhydrazyl) antioxidant capacity were quantified in plasma. Significant differences indicated that BPC-350 decreases MDA plasma concentration and protein carbonyls (*p* < 0.05). Additionally, a significant increase in the DPPH antioxidant capacity was observed in Meals 2 and 3 when compared to Meal 1 (*p* < 0.05). The results are discussed in terms of oxidative reactions that occur during digestion at the stomach level and the important effect of oxidative reactions that occur during the thermal processing of red meat.

## 1. Introduction

Polyphenols widely present in foods such as fruits, vegetables, and red wine have been proposed as key molecules associated with the beneficial effects of a Mediterranean diet [1–3]. Considerable evidence has demonstrated that foodstuffs or extracts with a high polyphenol content can induce protective effects against oxidative stress and inflammation in cells and animals [4–6]. Dietary intervention and postprandial studies in humans have also showed protective effects against oxidative stress and inflammation [7–11]. Oxidative stress has been closely associated with the pathogenesis of numerous chronic diseases as well as metabolic syndrome [12]. Plasma concentrations of oxidative stress markers such as malondialdehyde (MDA), 8-isoprostane, and isofurans are significantly increased in diabetic subjects as well as patients with cardiovascular diseases [13–15]. Additionally, plasma postprandial concentrations of MDA increase after high-fat meals. MDA can react through a Michael addition with glutathione, proteins, and nucleic acids, inducing cytotoxicity in cells and acting as a genotoxic agent [16, 17].

The postprandial oxidative response to eating depends on several factors including the chemical nature of the macronutrient intake [18, 19], the unsaturation degree of dietary fatty acids [20], the lipid intake dose [18], phytonutrient quantity [21], gender [22, 23], smoking habits [24], and race [25], among others. This oxidative response generates an increase in the plasma concentrations of MDA [26], lipid peroxides [27], protein carbonylation [20, 28], and hydrogen peroxide [20]. Postprandial oxidative stress induced by intake of high-saturated fatty acids can also alter the proteomic profile of peripheral blood mononuclear cells in patients with metabolic syndrome, which has been related to an increase in THBS-1 expression [29], a glycoprotein that promotes platelet aggregation [30]. This information underscores the importance of developing functional foods aimed at effectively reducing levels to the postprandial state [31].

Polyphenols present in red wine and roasted ground coffee effectively protect against postprandial oxidative stress, decreasing plasma concentrations of MDA after red meat cutlet intake [10, 32]. This effect has been explained by the ability of polyphenols to prevent oxidative reactions occurring during digestion primarily at the stomach level, where an acidic environment can enhance lipid peroxidation reactions [33].

Native Chilean berries possess high antioxidant activity and, among 120 species and varieties studied, native berries such as maqui* (Aristotelia chilensis)*, murta* (Ugni molinae)*, and calafate* (Berberis microphylla)* displayed the highest Oxygen Radical Absorbance Capacity (ORAC) antioxidant activities [34]. In human endothelial cell cultures, the addition of maqui, blackberry, or strawberry juice significantly protects cells from hydrogen peroxide-induced intracellular oxidative stress, with maqui and blackberry inhibiting more effectively than strawberry [35]. In addition to the in vitro antioxidant capacities, intracellular antioxidant responses are activated by berry polyphenols, including Chilean wild raspberries (*Rubus geoides* Sm.), which induced an increase in the intracellular glutathione content [4].

This work develops and characterizes a Chilean berry concentrate that consists of four berries produced in Chile, two of which are native species, with a high antioxidant capacity. In a randomized, crossover study, the effects of ingesting a dilute beverage prepared from the berry concentrate with a turkey meat burger prepared with or without 6% of the same concentrate were assessed on postprandial response, in terms of oxidative stress markers and antioxidant capacity.

## 2. Methods

### 2.1. Berry Concentrate Composition

The berry concentrate liquid mixture (BPC-350) was designed by the Center for Molecular Nutrition and Chronic Diseases, Pontificia Universidad Católica de Chile and provided by the Bayas del Sur S.A. Company (Purranque, Chile). The BPC-350 had a pH of 3.91 and 65°Bx and was prepared from the concentrate of four different berries, among them cranberry, blackberry, blueberry, raspberry, murta, and maqui, in a specific formulation to obtain a high antioxidant capacity mixture with good taste. Murta* (Ugni molinae)* and maqui* (Aristotelia chilensis)* are two native Chilean berries.

Good quality fruit was washed, sorted, and crushed with equipment specific to each operation. The juice obtained after pressing the crushed fruit mass was filtrated. For temporary preservation (inactivate enzymes and microorganisms) an operation with heat exchangers at ~80°C during 60 s was conducted. Concentrated juice production was performed by evaporation under a vacuum (0.5 bar residual pressure) at 40°C up to a concentration of 65°Bx, which assured preservation without further pasteurization.

### 2.2. Antioxidant Capacity and Polyphenol Determination in BPC-350

Antioxidant capacity was measured by ORAC and Ferric Reducing Antioxidant Power (FRAP) and determined according to Cao et al. [39] and Benzie and Strain [40], respectively. The ORAC was expressed as *μ*mol Trolox equivalents per g and FRAP was expressed as *μ*mol Fe^+2^ equivalents per g. Total polyphenol concentration was determined with Folin-Ciocalteu method [41] and was expressed as mg of gallic acid equivalents per g. Total monomeric anthocyanins were determined through the AOAC Official Method 2005.02 [42] and were expressed as mg of cyanidin 3-glucoside equivalents per g. Identification of the berry concentrate polyphenols was conducted by HPLC with UVA-visible detection at 280 nm, 360 nm, and 518 nm, comparing the retention times and UV-visible spectra of the compounds with pure standards. The polyphenol content was quantified by interpolation in a calibration curve built with the pure standards. This analysis was performed in three different polyphenol fractions: neutral, acid, and aqueous, according to the method originally reported by Salagoïty-Auguste and Bertrand [43] and modified for berry juice analysis by Miranda-Rottmann et al. [35]. In brief, neutral fraction was prepared by adjusting BPC-350 to a pH of 7.0 by adding 1 M NaOH and extracted in triplicate by agitation for 30 min in the dark with ethyl acetate. The organic phase was collected and evaporated to dryness with nitrogen and dissolved in HPLC-grade methanol (Merck, Darmstadt, Germany). The acid phase was collected from the previously obtained aqueous phase and the pH adjusted to 2.0 with 1 M HCl and extracted three times with ethyl acetate, as previously described. The organic phase obtained constituted the acid fraction and the remaining aqueous phase contained the anthocyanins. Neutral, acid, and aqueous fractions were analyzed by HPLC detecting at 360, 280, and 518 nm, respectively.

### 2.3. Test Meals and Beverage

Ground turkey leg meat was provided by Sopraval S.A (La Calera, Chile). The ground meat was mixed with vegetable oil (90% soya, 10% sunflower) at 6% w/w and salt at 0.25% w/w and then cooked as a burger in an electric oven for 20 min at 250°C. Once cold, the burgers were vacuum packed and stored at −20°C until use. Burgers with BPC-350 were prepared using the same recipe and included 6% w/w BPC-350 concentrate in the mix of ground meat, vegetable oil, and salt. The BPC-350 beverage was made with BPC-350 concentrate at 5% w/v, purified water, and sucralose as sweetener and was then pasteurized.

MDA levels were quantified in representative samples of turkey burgers according to the Csallany et al. protocol with modifications [44]. Briefly, 2 g of meat were homogenized in 10 mL of Milli-Q water using a tissue homogenizer for 30 s. Then 10 mL of trichloroacetic acid (TCA, 10%) was added, the solution was homogenized and centrifuged at 10000 rpm for 10 min at 4°C, and the supernatant was filtered. Of this solution, 700 *μ*L was treated with 500 *μ*L of 0.6% 2-thiobarbituric acid (TBA) solution and incubated at 90°C for 45 min, and the complex MDA-TBA_2_ was detected by HPLC using the methodology described below.

### 2.4. Subjects

Eleven presumably healthy male subjects between 18 and 35 years old were recruited through an advertisement placed at the Biological Sciences Faculty at the Pontificia Universidad Católica de Chile, Santiago, Chile. The inclusion criteria were males between 18 and 35 years old that agree to participate in the study and have read and signed the informed consent form. The exclusion criteria were (1) pharmacological treatment that would affect lipid profile, arterial pressure, carbohydrate metabolism, or plasma antioxidant profile; (2) diabetes mellitus, arterial hypertension, or dyslipidemias; or (3) chronic inflammatory diseases. Two subjects did not complete the study and were excluded from the analysis.

Subjects were evaluated through a medical interview to determine family antecedents and cardiovascular disease risk factors with special emphasis on evaluation of metabolic syndrome components, including arterial pressure and anthropometric parameters, among others. Additionally, each volunteer was evaluated through validated self-reported questionnaires, including Chilean Mediterranean Diet Index (Chilean-MDI), instrument developed and validated for the Center of Molecular Nutrition and Chronic Diseases of the Pontificia Universidad Católica de Chile, and physical activity, through the IPAQ (International Physical Activity Questionnaire) [45]. The subjects consisted of nine men with normal blood lipid and glucose levels with a mean age of 20 years and body mass index of 24.9 kg/m^2^ (further information in Table 1).

### 2.5. Bioethical Statement

This study was approved by the bioethics committee of the Biological Sciences Faculty of the Pontificia Universidad Católica de Chile and accomplished with the World Medical Helsinki Declaration.

### 2.6. Study Design, Treatments, and Procedures

A randomized type I clinical trial with a crossover design was conducted. The basic design involved administration of a test meal followed by blood sampling. Each subject consumed, in a random order, three different test meals on three different occasions separated by ≥1 week.

Subjects reported to the laboratory in the morning following a 12 h overnight fast. They were asked to exclude intake of creams and fried foods and to have no more than 1 cup of alcohol and not to smoke the day before each intervention. Test meals consisted of (a) Meal 1: 250 g of ground turkey leg meat burger and 500 mL of purified water (without gas); (b) Meal 2: 250 g of ground turkey leg meat burger and 500 mL of 5% BPC-350 beverage; (c) Meal 3: 250 g of ground turkey leg meat burger prepared with 6% BPC-350 and 500 mL of 5% BPC-350 beverage. A cannula was inserted into the vein of the forearm and a baseline (time 0) fasting blood sample was collected in corresponding tubes. The subjects then ate a test meal within 20 min. Blood samples (15 mL) were drawn every hour for 6 hours after consumption. Analyses including biochemical profile, lipid profile, and glycaemia were performed at the Clinic Hospital of the Pontificia Universidad Católica de Chile. Analyses of oxidative stress markers (MDA and protein carbonyls), antioxidant capacity (FRAP, and DPPH), and Vitamin C were performed at the Molecular Nutrition and Chronic Diseases Center of the Pontificia Universidad Católica de Chile. Vitamin C was determined by spectrophotometry according to Day et al. [46]. During the analysis the researchers were blinded to exclude possible bias.

### 2.7. Malondialdehyde Determination

MDA was quantified according to the Templar et al. [47] protocol with some modifications. Briefly, human plasma was deproteinized using 5% TCA and supernatant was treated with fresh 0.6% TBA and incubated for 45 min at 90°C. The mixture was cooled at room temperature (25°C) and 120 *μ*L was injected in the HPLC. The MDA concentration was determined by interpolating the area of the peak corresponding to the adduct MDA-TBA_2_ of the sample into a MDA calibration curve prepared through acid hydrolysis of 1,1,3,3-tetraethoxypropane. The HPLC measurements were performed using a reverse-phase HPLC Merck-Hitachi 7000 series (Merck-Hitachi, Darmstadt, Germany) equipped with an autosampler device. The separation was reached using an Inertsil ODS-3 column (GL Sciences, Tokyo, Japan) and a mobile phase composed of a 50 mM sodium phosphate buffer, pH 7 (65%) and methanol (35%). The detection was conducted using a UV-visible photodiode array detector and a fluorescence detector (*λ*_EXC_ = 515 nm; *λ*_EM_ = 550 nm). The fluorescence chromatograms were only used for MDA analysis.

### 2.8. Protein Carbonyl Content

Determination of carbonyl groups in oxidized plasma proteins was assessed by derivatization with 2,4-dinitrophenylhydrazine, according to the method of Levine et al. [48]. Spectrophotometric measurement of plasma reactive carbonyl derivatives was performed and calculated using the extinction coefficient of 2,4-dinitrophenylhydrazine-reactive carbonyl derivatives at 370 nm = 22 × 10^3^ L mol^−1^ cm^−1^ and expressed as *μ*mol/mg of proteins [48].

### 2.9. Antioxidant Capacity Determination

Antioxidant activity in human plasma was measured using the DPPH method adapted from Chrzczanowicz et al. [49] with some modifications. Briefly, plasma samples (100 *μ*L) were deproteinized by adding 300 *μ*L of acetonitrile and centrifuging for 10 min (4°C, 9500 ×g). Supernatant was immediately collected and 50 *μ*L was transferred to a microplate, then 100 *μ*L of 0.15 mM DPPH was added, and the absorbance was read at 517 nm during a 30 min incubation period at room temperature (25°C).

A negative control was conducted with acetonitrile (at the same concentration) and the experiments were performed in triplicate. A calibration curve was built with Trolox where the absorbance values were interpolated and the results were expressed as Trolox equivalents. To distinguish and compare the differences between each meal, DPPH-DPPH_0_ was calculated, where DPPH means the Trolox equivalents at any time and DPPH_0_ means the Trolox equivalents at time zero. The FRAP was determined in plasma obtained from heparin tubes, according to Benzie and Strain [40].

### 2.10. Statistical Analysis

Subject descriptive characteristics are presented as median and range. Oxidative stress (MDA and protein carbonyls) and antioxidant status biomarkers (DPPH, FRAP, and Vitamin C), were analyzed using a 3 (meal) × 7 (time) repeated measures analysis of variance (ANOVA). Significant interactions and main effects were further analyzed using Tukey's post hoc tests. For each biomarker, the area under the curve (AUC) was calculated using the trapezoidal method. One-Way ANOVA and paired Student's* t*-tests were used to analyze statistical differences between AUCs. Student's* t*-test for independent samples was used to analyze statistical differences between MDA in uncooked and cooked meats. Data processing and statistical analyses were performed using the SPSS statistical software package, version 17.0 (SPSS Inc., Chicago, IL) and R (Team RC. R: A Language and Environment for Statistical Computing. 2014.). *p* ≤ 0.05 was considered statistically significant.

## 3. Results

### 3.1. BPC-350 Characterization

The BPC-350 antioxidant capacity determined by ORAC and FRAP assays was 453.74 ± 19.95 *μ*mol Trolox equivalents per g and 313.4 ± 16.9 *μ*mol equivalents Fe^+2^ per g, respectively (Table 2). Total phenolics determined by Folin-Ciocalteu were 25.56 ± 1.08 mg gallic acid equivalents per g (GAE/g) and total anthocyanins determined by the pH differential method were 5.62 ± 0.19 mg cyanidine 3-glucoside equivalents per g (Table 2). The total quantification of the main compounds present in BPC-350 in terms of the three polyphenol families analyzed was anthocyanins (5,170 *μ*g/g) > phenolic acids (411.7 *μ*g/g) > flavonoids (263.4 *μ*g/g).

The pasteurized BPC-350 beverage made with BPC-350 concentrate at 5% w/v, purified water, and sucralose as sweetener had an ORAC of 14.23 ± 0.19 *μ*mol Trolox equivalents per g, total phenolics of 1.39 ± 0.05 mg GAE/g, and total anthocyanins of 0.18 ± 0.03 mg cyanidine 3-glucoside equivalents per g (Table 2).

**Figure 1:**
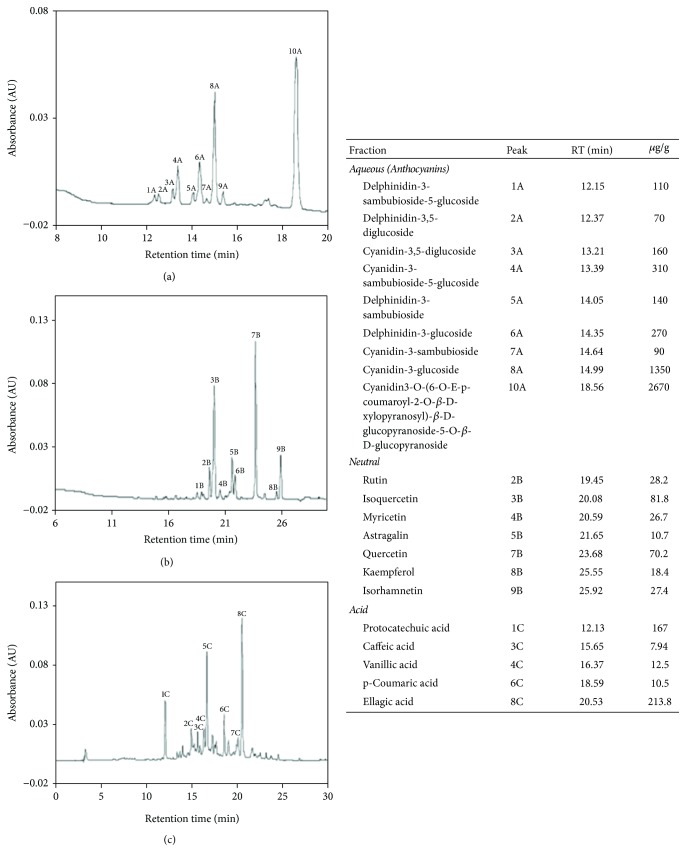
Identification and quantification of polyphenolic compounds present in BPC350 by HPLC-UV-Vis chromatography. Panel (a) shows the chromatographic profile, detecting at 360 nm (neutral fraction). Panel (b) shows chromatographic profile detecting at 360 nm (acid fraction). Panel (c) shows chromatographic profile detecting at 518 nm (aqueous fraction). Tables show identification and quantification of chromatographic peaks.

The HPLC analysis of polyphenols from neutral, acid, and aqueous fractions of BPC-350 are displayed in Figure 1. Through HPLC analysis performed by comparing the retention times and UV-visible spectra with pure standards, it was possible to identify 7 flavonoids, 4 phenolic acids, and 10 anthocyanins present in neutral, acid, and aqueous fractions, respectively (Figure 1). The principal components of the anthocyanin family were identified as Cyanidine 3-glucoside and Cyanidin3-O-(6-O-E-p-coumaroyl-2-O-*β*-D-xylopyranosyl)-*β*-D-glucopyranoside-5-O-*β*-D-glucopyranoside. Alternatively, isoquercetin and ellagic acid were the principal components of the neutral and acid fractions, respectively (Figure 1).

### 3.2. Test Meal Analysis

**Figure 2:**
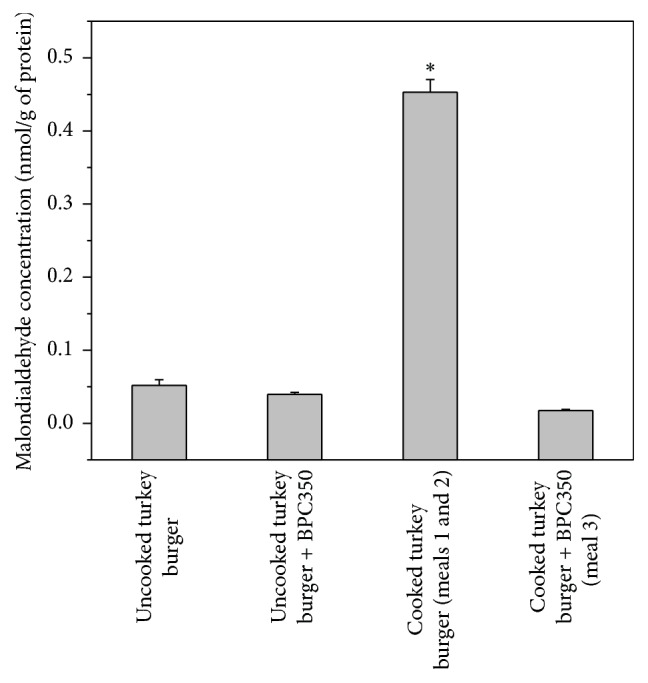
Malondialdehyde content in experimental foods used to perform the crossover study. Malondialdehyde concentrations were quantified in uncooked and cooked turkey burgers prepared with or without BPC-350 at 6% (*N* = 3). Data were expressed as the mean ± SD. *∗* indicates significant differences when compared to uncooked turkey burger, uncooked turkey burger + BPC-350, and cooked turkey burger + BPC-350 (*p* < 0.05).

To determine whether BPC-350 could affect MDA generation during thermal meat processing, the MDA concentration was quantified in turkey burger samples. Control turkey burgers processed thermally (cooked) presented MDA concentration 8.7 times higher than uncooked burgers (Figure 2). However, turkey burgers prepared with 6% BPC-350 concentrate did not increase MDA concentration after cooking (Figure 2).

### 3.3. Postprandial Study

**Figure 3:**
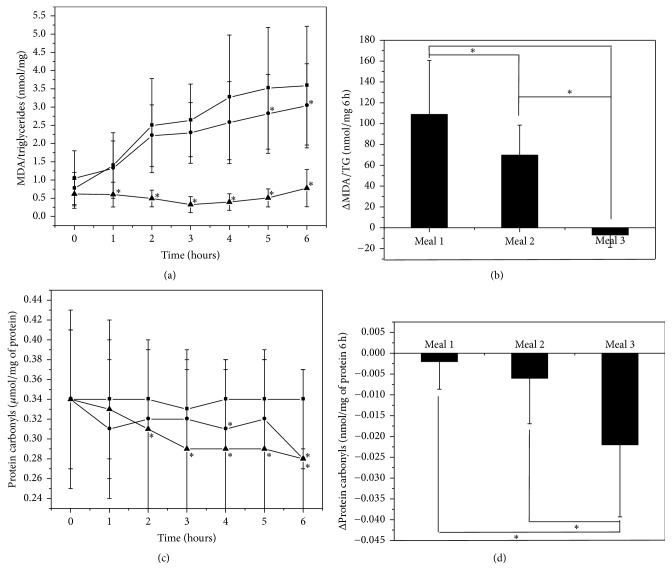
Plasma postprandial malondialdehyde and protein carbonyls in human healthy subjects after the intake of three different meals: ■ Meal 1 (turkey burger + water); ● Meal 2 (turkey burger + 5% BPC 350 beverage); ▲ Meal 3 (turkey burger prepared with 6% BPC-350 + 5% BPC-350 beverage). Panels (a) and (c) show the time profile of malondialdehyde corrected by triglycerides and protein carbonyls concentrations after the intake of the three different meals, respectively. Panels (b) and (d) show the area under the curve of the different time profiles corresponding to three different meals for malondialdehyde and protein carbonyls, respectively. Data were expressed as the mean ± SD. In Panels (a) and (c), *∗* shows significant differences for each time compared to Meal 1 (*p* < 0.05). In Panels (b) and (d), *∗* shows significant differences between meals (*p* < 0.05).

To determine the effect of the Chilean berry concentrate BPC-350, a type I clinical trial with a crossover design was performed with 11 healthy male subjects. On different days, after fasting overnight, the volunteers consumed the following meals: 250 g of oven-cooked turkey burger (11.25 *μ*mol of MDA) and 500 mL of water (Meal 1); similar burger (11.25 *μ*mol of MDA) and 500 mL of 5% BPC-350 beverage (639 mg GAE of polyphenols) (Meal 2); or 250 g of oven-cooked turkey burger with 6% BPC-350 concentrate (383 mg GAE of polyphenols, 0.5 *μ*mol MDA) and 500 mL of 5% BPC-350 beverage (639 mg GAE of polyphenols) (Meal 3). Postprandial changes in glucose, triacylglycerol, MDA, protein carbonyls, DPPH, FRAP, and Vitamin C were measured over the 360 min (6 h) experimental period at 60 min intervals after meal consumption. No differences were found in glucose and triacylglycerol patterns, nor in the values of the areas under the curve between the meals (data not shown). The time profile of MDA per mg of triacylglycerol can be observed in Figure 3(a). The intake of Meals 1 and 2 significantly increased the mean values (*N* = 11) of MDA per mg of triacylglycerol during study time, reaching values ~5 and ~3 times higher than basal levels for Meals 1 and 2, respectively, which remained high after 6 hours (Figure 3(a)). However, comparing postprandial changes in MDA per mg of triacylglycerol at 5 h and 6 h, Meal 2 showed significantly lower concentrations of MDA per mg of triacylglycerol than Meal 1 (*p* < 0.05). Meal 3 did not significantly increase (*p* > 0.05) the MDA content of plasma in comparison to the baseline (time 0). Significant differences over the entire time range analyzed (6 hours after intake) were found when compared with Meals 1 or 2; this treatment completely prevented MDA accumulation in plasma.

The mean value of the area under the curve of the time profile of volunteer MDA/TG for the three meals is displayed in Figure 3(b). Compared to Meal 1, the mean value of the area under the curve of MDA/TG for Meals 2 and 3 presented a statistically significant reduction (*p* < 0.05). Meal 3 also presented a statistically significant decreased area under the curve compared with Meal 2 (*p* < 0.05).

The time profile of protein carbonyls after intake of the three experimental meals is shown in Figure 3(c). No significant changes in protein carbonyls were observed after intake of Meal 1 in the time ranges analyzed. However, decreases in the protein carbonyl concentrations were observed for Meals 2 and 3. When compared with Meal 1, statistically significant differences were found at 4 h and 6 h with Meal 2 and at 2 h to 6 h with Meal 3. The area under the curve of the time profiles for proteins carbonyls (Figure 3(d)) displayed significant decrease after Meals 2 and 3 compared to Meal 1.

**Figure 4:**
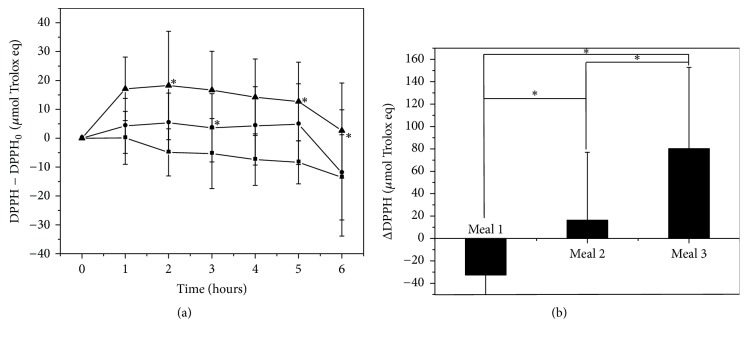
Changes in the plasma antioxidant activity determined by means of DPPH method before and after the intake of three different meals. ■ Meal 1 (turkey burger + water); ● Meal 2 (turkey burger + 5% BPC 350 beverage); ▲ Meal 3 (turkey burger prepared with 6% BPC-350 + 5% BPC-350 beverage). Panel (a) shows the time profile of changes in DPPH antioxidant activity. Panel (b) shows the area under the curve of the time profiles, corresponding to the three different meals. Data were expressed as the mean ± SD. In Panel (a), *∗* shows significant differences for each time compared to Meal 1 (*p* < 0.05). In Panel (b), *∗* shows significant differences between meals (*p* < 0.05).

To determine the effect of BPC-350 on antioxidant activity, human plasma was evaluated with the FRAP and DPPH radical scavenging capacity. Only the DPPH antioxidant assay presented statistically significant differences between meals. The changes in the scavenging capacity of DPPH radical activity during the study are exhibited in Figure 4(a). Meal 1 consumption decreased the antioxidant activity of volunteers' plasma along the time range (*p* < 0.05) (Figure 4(a)). After intake of Meal 2, plasma antioxidant activity remained constant until 5 h. Alternatively, Meal 3 consumption increased plasma antioxidant activity 1 hour after intake, which remained high and only decreased after 6 hours (Figure 4(a)). When compared with Meal 1, statistically significant differences were found at 3 h with Meal 2 and at 2 h, 5 h and 6 h with Meal 3.

The area under the curve for changes in volunteer DPPH scavenging activity after meals is presented in Figure 4(b). The area under the curve of DPPH time profiles for Meals 2 and 3 displayed a significant increase compared with Meal 1 (*p* < 0.05).

**Figure 5:**
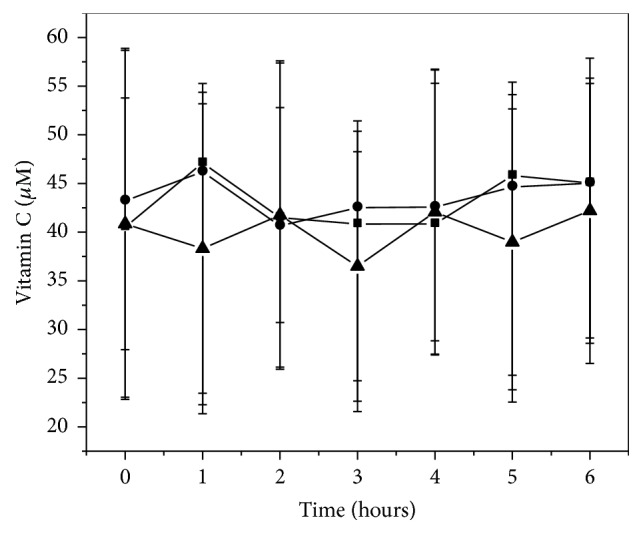
Plasma postprandial Vitamin C in human healthy subjects after the intake of three different meals: ■ Meal 1 (turkey burger + water); ● Meal 2 (turkey burger + 5% BPC 350 beverage); ▲ Meal 3 (turkey burger prepared with 6% BPC-350 + 5% BPC-350 beverage). Data were expressed as the mean ± SD.

The time profile of Vitamin C after intake of the three experimental meals is shown in Figure 5. Vitamin C did not present statistically significant differences between meals.

## 4. Discussion

Numerous epidemiologic and experimental studies have provided significant evidence regarding the beneficial effects of regular consumption of fruits and vegetables, which is associated with a high polyphenol content [50–52]. Red wine is one of the most studied and characterized beverages, generally known for having a high polyphenol content, which is concomitant with a high antioxidant capacity [3, 9, 53]. Gorelik et al. demonstrated that one of the functional properties of red wine is the ability to reduce postprandial oxidative responses (in terms of MDA quantities) in humans, produced by the intake of red meat [9]. This fact has been explained by the inhibition of oxidative reactions that occur at the stomach level that could be catalyzed by Fe^+2^ through a Fenton reaction, among others [32, 33]. However, medical recommendation of wine consumption possesses the limitations inherent to alcoholic beverages and could not be implemented as a public health strategy to reduce chronic diseases. In this sense, this work aimed to elaborate a nonalcoholic drink with a high antioxidant capacity that could be similar to the one found in red wine and to determine whether it could exert protective effects in the postprandial response in terms of oxidative stress, and antioxidant capacity in healthy humans. Considering the high antioxidant capacity [34] and evidence supporting the health effects of berry consumption [35, 54], the Chilean berries were selected for this study. The effects of 5% w/v BPC-350 beverage were assessed through a crossover study performed with 11 healthy male volunteers, determining the responses in terms of oxidative stress markers (MDA and protein carbonyls) and antioxidant status biomarkers (DPPH, FRAP, and Vitamin C) induced by the intake of three different meals.

The analysis of BPC-350 antioxidant capacity was evaluated using two different antioxidant assays: ORAC and FRAP (Table 2). These results indicate a high antioxidant capacity in comparison to other berries [34, 55]. Anthocyanins are the main phenolic compounds reported in native Chilean berries such as maqui* (Aristotelia chilensis)* or calafate* (Berberis microphylla)* [35, 55, 56]. The analysis of BPC-350 phenolic content by HPLC (Figure 1) indicated that anthocyanins are the primary polyphenols present in BPC-350 (88.5%), and flavonoids and phenolic acids account for 4.5% and 7%, respectively. The primary anthocyanins present in BPC-350 were cyanidin3-O-(6-O-E-p-coumaroyl-2-O-*β*-D-xylopyranosyl)-*β*-D-glucopyranoside-5-O-*β*-D-glucopyranoside and cyanidin 3-glucoside (Figure 1), which are present in high amounts in berries such as* Sambucus nigra* [57] and* Ribes* (*magellanicum* and* cucullatum*) [56], respectively. In red wine, anthocyanins represent ~70%, and are primarily malvidin glucosides but also contain a quantity of cyanidin 3-glucoside [58]. The reactivity of the primary anthocyanins against numerous reactive oxygen species is similar in the 5% w/v BPC-350 beverage and red wine [59], despite this beverage presenting lower total polyphenols content and antioxidant activity than an average red wine [60]. This formulation was created to obtain a high quality polyphenols beverage with good taste.

Volunteers participating in this study were considered normal according to international recommendations and no subjects with diabetes or metabolic syndrome were detected in this population (Table 1) [38]. This allows the comparability of data obtained from the postprandial responses analysis since alterations in glycemia, triacylglycerides, oxidative stress markers, and serum antioxidant enzyme activity have been reported in cases with diabetes and metabolic syndrome [61–64].

When volunteers ate the turkey burger with water, plasma MDA/TG levels rose, but when volunteers drank 5% w/v BPC-350 beverage instead of water they exhibited a 35% reduction in the area under the curve of plasma MDA/TG concentration during the 6 hour after meal period. This beverage diminished MDA accumulation in plasma. Interestingly, intake of the turkey burger with water did not change carbonyls in plasma proteins. However, the 5% w/v BPC-350 beverage produced a significant decrease in plasma protein carbonyl concentration. These results indicate that the 5% w/v BPC-350 beverage, under the scheme of this study, prevented lipid peroxidation and the consequent formation of MDA and MDA-protein adduct that occurs during digestion at the stomach level [65].

These results agree with the work of Gorelik et al., which analyzed the effect of red wine on postprandial oxidative stress modulation [9]. They also found decreased plasma MDA levels after intake of 250 g of red meat turkey cutlets soaked in red wine concentrate after heating plus 200 mL of wine, compared to eating 250 g of red meat cutlets plus water [9]. However, the magnitude of the red wine effect was double that of the 5% w/v BPC-350 beverage even with a similar quantity of polyphenols. The difference could be explained by the diversity in polyphenol quality, especially on the capacity to quench lipid oxidation. Alternatively, turkey burgers prepared with vegetable oil (60% polyunsaturated fatty acids) at 6% w/w are susceptible to oxidation, which could result in a polyphenol quantity insufficient for stopping the oxidation reactions. Additionally, due to the suspension of polyphenols in 500 mL of 5% w/v BPC-350 beverage, it is possible that the beverage passed from the stomach to the intestine too quickly and lacked sufficient contact with the meat to act as an antioxidant.

Reports indicate that MDA derived from meat consumption modified low-density lipoprotein (LDL) in vivo, and this modification was directly dependent on the increased plasma MDA level following a meal [33]. Aldehydes such as MDA may react with lysine residues in the LDL apo B-100 moiety, resulting in a decreased apo B-100 affinity for the LDL receptor [66]. Also, albumin and plasma proteins react with MDA or other electrophiles produced during lipid oxidation [67]. This reaction has been studied in vitro, demonstrating than the rate of generation of protein carbonyl is low at physiological pH [68]. This could explain in part the absence in protein carbonyl increase after the intake of Meal 1, even when this meal increased significantly the plasma levels of MDA.

Rising evidence supports that compounds like MDA can have specific signaling roles inducing adaptive responses driven to decrease oxidative damage and improve antioxidant defenses [69]. In fact, it has been reported an increase in the plasma levels of thiols after the intake of high-fat meals [70]. This endogenous antioxidant response to postprandial oxidative stress, proposed as part of a protein oxidation defense [70], involves the activation of the transcription factor Nrf2, being the master regulator factor [69]. Also, in vitro studies have demonstrated that carbonylated proteins can be reduced by a nonenzymatic reaction with sulfhydryl groups present in cysteine and glutathione [71]. So an increase in plasma thiols after intake of Meal 1 could prevent protein oxidation according to a physiological mechanism of protection. However, we did not measure plasma thiols so this is a hypothesis that should be demonstrated.

Similar reasons as Meal 1 could explain in part the decrease in protein carbonyl after eating the turkey burger with the 5% w/v BPC-350 beverage (Meal 2). But in this case plasma MDA level was significantly lower than after intake Meal 1. Lower plasma MDA quantity means less reaction with protein and, therefore, less carbonyl in plasma protein considering the relatively low reaction rate between proteins and MDA. The proteins synthetized de novo could contain less carbonyl than older proteins in the basal state.

On the other hand, polyphenolic enriched extracts of the Chilean native berry Rubus Geoides have shown to increase glutathione levels in AGS cells [4]. Also, in Wistar rats an increase in plasma glutathione after intake of a mixture of grape seed proanthocyanidin and docosahexaenoic acid has been reported [72]. In this sense, the intake of BPC-350 rich in polyphenols could increase plasma glutathione and promote decarbonylation of protein. Evidence suggests that polyphenols may induce cellular defense genes by a mechanism that includes Nrf2 activation [4, 73, 74]. As mentioned before, plasma thiols and glutathione were not measured in this study, so additional experiments are necessary to confirm our hypothesis.

MDA, a red meat-derived aldehyde, can interact and modify LDL in plasma, possibly enhancing atherosclerotic plaque production [33]. Ahotupa et al. found that food lipid peroxides are incorporated into serum triglyceride-rich lipoproteins and LDL, directing the lipid peroxide flow towards peripheral tissues [75]. They propose that the specific atherosclerosis-related effects of serum lipoproteins are not explained only by cholesterol transport but also from the transport of atherogenic lipid peroxides [75].

Meat consumption with plant derived polyphenols (e.g., red wine or coffee polyphenols) can prevent the appearance of MDA in plasma and LDL modification [9, 10]. Therefore, we suggest that the harmful consequences of red meat product consumption might be partially diminished by simultaneous polyphenol addition to meals with red meat. Polyphenol treatment of red meat during preparation (e.g., cooking and processing) may also significantly contribute to the prevention of hazardous and deleterious effects of red meat products.

Other studies have demonstrated the effect of polyphenols, primarily wine, on postprandial oxidative stress [76]. Natella et al., who used a test meal consisting of “Milanese” meat and fried potatoes, observed that intake of the meal with 400 mL of red wine provoked a significant increase in total plasma antioxidant capacity and a reduction in the postprandial increase of LDL susceptibility to oxidation [77]. In a similar study, Di Renzo et al. used a McDonald's Meal (N.1 Big Tasty Bacon Sandwich and N.1 small French Fries) with 250 mL of red wine, which resulted in lower (*p* < 0.05) values of postprandial ox-LDL than meal consumption without red wine [78].

When MDA plasma concentrations were analyzed after intake of Meal 3, no significant differences were observed when comparing MDA basal levels (time 0) with those produced 6 hours after intake, indicating the inhibitory effect of plasma MDA produced by this meal. The mean values of the area under the curve indicate a complete reduction of MDA absorption compared with the response after intake of Meals 1 and 2. In agreement with this information, we determined that the MDA quantity of Meal 3 (turkey burger prepared with BPC-350 concentrate and cooked) was 22.5 times less than the MDA quantity of Meal 1 and 2 (turkey burger prepared without BPC-350 and cooked).

These data suggest that the strong effect observed for Meal 3 was due to the inhibition capacity of lipid peroxidation reactions by BPC-350 concentrate when the turkey burger was thermally processed. There was a dual protective effect produced by the inhibition of lipid peroxidation reactions, occurring at the stomach level and during thermal food processing. This fact is also consistent with in vitro studies that have demonstrated anthocyanins ability to inhibit lipoperoxidation [79, 80].

The effect of BPC-350 intake on the antioxidant capacity was determined in plasma by FRAP and DPPH measurements. Significant differences for the plasma antioxidant capacity were found when the different meals were compared using DPPH values. Considering that human plasma was deproteinized before DPPH antioxidant activity determination, to ensure reproducibility [49], any effect associated with scavenging of DPPH radical due to changes in protein expression can be discarded. This implies that the increase in the antioxidant capacity determined by DPPH after intake of Meals 2 and 3 could be due to low molecular weight molecules, including polyphenols, Vitamin C, and urate. We did not observe significant differences between meals comparing Vitamin C plasma concentrations curves either point to point or calculating the area under the curve. Therefore, considering that BPC-350 do not contain Vitamin C, the increase in the antioxidant activity induced by BPC-350 intake (especially in Meal 3) could be attributed to other compounds present in this concentrate or synthesized in the organism after consumption. Human plasma analysis after intake of blueberries indicated the presence of 19 out of 25 anthocyanins originally present in the fruit [81], which suggests the possibility of anthocyanins contribution to the antioxidant activity found in this study. However, the contribution of phenolic acids cannot be discarded, including those produced from anthocyanin metabolism occurring in the liver and microbiota. In this sense, a study of urate levels after intake of BPC-350 could clarify the mechanisms associated with the increased antioxidant activity observed in this study.

The intake of Meal 1 diminished the antioxidant capacity of plasma measured as DPPH, according to the increase in MDA plasma concentrations. This could be explained by the utilization of endogenous antioxidants (able to react with DPPH radical) such as urate and glutathione [82, 83], due to hydroperoxide absorption, which promotes lipid oxidation. The postprandial state induces immediate oxidative stress that triggers atherogenic changes including inflammation, endothelial dysfunction, hypercoagulability, and sympathetic hyperactivity [84].

Postprandial oxidative stress, which occurs after eating meat fat, is associated with a higher risk for atherosclerosis, diabetes, and obesity. In Western societies, a significant portion of the day is spent in a postprandial state. Lipid hydroperoxides present in the diet are absorbed, producing endothelium-dependent vasodilation. Postprandial oxidative stress is attenuated when dietary antioxidants are supplied with a meal rich in oxidized or oxidizable lipids. Ingestion of dietary polyphenols, for example, from wine, cocoa, or tea, improves endothelial dysfunction and lowers LDL lipid susceptibility to oxidation. Polyphenols affect endothelial function not only as antioxidants but also as modulatory signaling molecules.

The consumption of high-fat and high-iron potentially prooxidant foods such as red meat produced postprandial oxidative stress, as detected by the increment in plasma MDA and the reduction in plasma antioxidant capacity. The intake of food- or beverage-derived polyphenols with the meal prevented plasma oxidative stress, as evidenced by this work and those of other researchers [9, 76, 85].

The Mediterranean diet is currently considered a healthy dietary pattern. It includes a great variety of foods, which are eaten in moderation and within a positive social environment. The way of cooking food in Mediterranean cuisine has been associated with lower cardiovascular risk. The basis of Mediterranean dishes is the sauté of onion, garlic, and tomato in olive oil; this source of antioxidants is used to flavor vegetables such as zucchini, eggplant, potatoes, and haricot verts; cereals such as rice or pasta; legumes such as beans or chickpeas; and even meat, poultry, or fish. A wide variety of spices and condiments like lemon, vinegar, parsley, mint, oregano, herbs, cinnamon, and many others, is used for seasoning salads and different preparations. Polyphenols widely present in characteristic Mediterranean foods such as fruits, vegetables, and wine red and the way to use them in the Mediterranean cuisine could explain the beneficial effects of Mediterranean diet.

The results obtained in this study indicate the usefulness of a berry-based drink to decrease postprandial oxidative stress. Our results emphasize the effectiveness of a berry concentrate for inhibiting lipoperoxidation reactions occurring at the stomach level but primarily during thermal treating of foods. The way in which food is prepared is critical to our health.

## Figures and Tables

**Table 1:** Anthropometric, clinical, and biochemical characterization of subjects participating in this study (*N* = 11).

Parameter	Median	Range	Reference values
Age (year)	20.2	18.7–27.3	
Waist circumference (cm)	87.4	74.0–100.5	<102^*∗*^
BMI (kg/cm^2^)	24.6	20.7–29.4	18.5–24.9^*∗∗*^
Systolic BP (mmHg)	117.5	112.5–130.0	≤130^*∗*^
Diastolic BP (mmHg)	70.0	57.5–90.0	≤85^*∗*^
Fasting glucose (mg/dL)	84	75–95	≤100^*∗*^
Triglycerides (mg/dL)	71	37–193	≤150^*∗*^
Total cholesterol (mg/dL)	150	113–177	<200^*∗*^
HDL (mg/dL)	49	32–59	>40^*∗*^
LDL (mg/dL)	93	51–105	<130^*∗*^
GGT (U/L)	18	8–27	9–31°
SGOT (U/L)	21	15–26	5–40°
Leukocytes	5.9	5.0–8.5	4–11°

^*∗*^Cutoff points for metabolic syndrome components according to NCEP-ATPIII definition [36].

^*∗∗*^BMI range for normal weight subjects [37].

°Normal range for males aged 18 to 35 years [38].

**Table 2:** Concentration of total polyphenols, total anthocyanins, and antioxidant capacity of BPC-350 and 5% BPC-350 beverage.

Assay	BPC-350 (mean ± SD)	5% BPC-350 beverage (mean ± SD)
ORAC (*µ*mol Trolox eq/g)	453.74 ± 19.95	14.23 ± 0.19
Total polyphenols (mg GAE/g)	25.56 ± 1.08	1.39 ± 0.05
Total anthocyanins (mg CE/g)	5.62 ± 0.19	0.18 ± 0.03

GAE: gallic acid equivalents; CE: cyanidine 3-glucoside equivalents.
